# Gerotherapeutic compound picolinic acid supports locomotor function and bone health in aged zebrafish

**DOI:** 10.1093/geroni/igaf122.532

**Published:** 2025-12-31

**Authors:** Hassan Rammal, Dafna Perry, Daniel Rivas, David Karasik, Gustavo Duque

**Affiliations:** McGill University, Montreal, Quebec, Canada; Azrieli Faculty of Medicine, Bar-Ilan University, Bar-Ilan, Hefa, Israel; McGill University, Montreal, Quebec, Canada; Azrieli Faculty of Medicine, Bar-Ilan University, Bar-Ilan, Hefa, Israel; McGill University, Montreal, Quebec, Canada

## Abstract

**Objectives:**

This study aimed to evaluate the impact of picolinic acid (PIC), a metabolite derived from tryptophan, on age-related tissue regeneration and physical decline in zebrafish. Additionally, it examined changes in whole-body mass index (WB-BMI) as an indicator of musculoskeletal aging.

**Methods:**

Siblings born in August 2022 were randomly assigned to four groups at age 20 mo: (1) PIC in water (25 mg/kg/day), (2) PIC in water + 25 mg/kg oral gavage, (3) PIC in water + 75 mg/kg oral gavage, and (4) control (system water + gavage) for eight weeks. The treatment groups consisted of 15 fish. At week 7, swimming performance was recorded over a 30-minute period. Caudal fins were amputated for regeneration analysis. In week 8, fish were euthanized for whole-body micro-CT and for β-galactosidase (X-gal) staining to evaluate cellular senescence. The Mann-Whitney U test was used to compare WB-BMI between groups.

**Results:**

Group 2 showed the highest swimming speed (44.5 m/min), followed by Group 1 (34.7 m/min) (p = 0.01 and p = 0.09, respectively) than the control group (31.7 m/min). WB-BMI was decreasing over the duration of the experiment in all 4 groups, with Group 1 maintaining the highest BMD compared to the control (non-significant, p > 0.5). Regeneration in Groups 2 and 3 was more advanced than in Group 1, with stronger staining by β-galactosidase.

**Conclusion:**

PIC at moderate doses supports locomotor function and overall health in aged zebrafish. It may influence regenerative outcomes through mechanisms involving cellular aging.

